# Exposure to, understanding of and interest in interventional radiology among Pakistani medical students: a cross-sectional study

**DOI:** 10.3389/fmed.2023.1226294

**Published:** 2023-10-16

**Authors:** Muneeb Chattha, Muhammad Junaid Tahir, Ahmad Zia, Maha Chattha, Waleed Tariq, Muhammad Faiq Masood, Salman Sani, Zohaib Yousaf, Mohammed Mahmmoud Fadelallah Eljack, Muhammad Sohaib Asghar

**Affiliations:** ^1^Department of Medicine, Foundation University Medical College, Rawalpindi, Pakistan; ^2^Department of Radiology, Pakistan Kidney and Liver Institute and Research Center (PKLI & RC), Lahore, Pakistan; ^3^Department of Radiology, Army Medical College, Rawalpindi, Pakistan; ^4^Department of Medicine, Mayo Hospital, Lahore, Pakistan; ^5^Department of Surgery, Lahore General Hospital, Lahore, Pakistan; ^6^Department of Medicine, Jinnah Hospital, Lahore, Pakistan; ^7^Department of Medicine, Tower Health, Reading, PA, United States; ^8^Department of Medicine, Faculty of Medicine and Health Sciences, University of Bakht Alruda, Ad Duwaym, Sudan; ^9^Department of Medicine, Mayo Clinic, Rochester, NY, United States

**Keywords:** medical specialty, awareness, knowledge, radiology, future career

## Abstract

**Background:**

Medical students need more awareness regarding minimally invasive image-guided procedures carried out by interventional radiological approach. This study analyzed the knowledge and attitudes of medical students regarding interventional radiology (IR) and the factors influencing their decision to choose IR as a specialty in the future.

**Methods:**

A cross-sectional, web-based study was conducted among medical students across Pakistan. The data were collected from October 14, 2021, to November 14, 2021. The questionnaire included demographic variables, exposure, interest, and self-reported knowledge of IR, interventions, instruments utilized in IR, and the responsibilities of the interventional radiologist. Variables affecting the possible choice of IR as a future career were analyzed using logistic regression analysis.

**Results:**

The median age was 22 years, with a male predominance. 65.5% exhibited an interest in radiology, and 20.2% in IR. The majority, 83.5%, perceived IR. As having good to adequate prospects. Male participants preferred IR more as compared to females. Participants willing to attend IR rotation and had an excellent view of IR as a specialty had higher propensity towards IR as a future career than their counterparts. The majority opted for IR as a better-paying job with lots of intellectual stimulation and career flexibility.

**Conclusion:**

IR is a demanding specialty with rigorous routines but reasonable monetary compensation. Lack of infrastructure and low numbers of trained specialists limit medical students’ exposure to IR in developing health economies like Pakistan. Clinical rotations in IR departments would help raise awareness about the field and bridging this gap.

## Introduction

The need for minimally invasive image-guided interventions has proven its effectiveness across the modern world in terms of better patient outcomes, reduced mortality, morbidity, and length of hospital stay in the United States (US) and European healthcare systems (1). It has positively shaped healthcare economics by reducing the cost of care by reinforcing the day case procedures (1). The demand for interventional radiology (IR) procedures is surpassing the availability of qualified specialists, particularly in the United States, Europe, and the Middle East (1, 2). This issue also holds true for the Southeast Asian region, where research and advancements in the field are actively progressing (3–5).

IR is an image-guided procedural domain of radiology. It is “keyhole surgery with x-ray vision.” The vision is achieved by incorporating modalities like ultrasonography, x-rays, and CT scan, resulting in minimally invasive procedures, shorter hospital stays, and fewer complications (6).

Due to low awareness levels, IR is a nascent field rarely chosen by medical students in Pakistan. Very few centers are providing formal training in the country (7). Lack of infrastructure, lack of knowledge regarding IR, and shortage of existing trained IR specialists are part of the problem. There is also a need for more exposure to IR during medical school (2, 5, 8). The medical students get hands-on experience during general surgery or internal medicine rotation but exposure to radiology in general and IR, in particular, is limited (1). There is a lack of role models for students and a general perception that radiologists are antisocial (9, 10). Cardiology and vascular surgery involving imaging and catheterization procedures usually get more interest from students (11).

IR in Pakistan is in the early phase with few training opportunities. This study aims to assess the exposure, interest, and understanding of various components of IR among medical students in Pakistan.

## Materials and methods

### Study design, sample size, and data collection

This is a cross-sectional study targeted towards medical students in Pakistan. A minimum sample size of 385 was estimated using the online Raosoft® sample size calculator, using a 95% confidence interval, 50% response distribution, 5% margin of error, and an estimated population size of 20,000 (12). The online Google forms® were used for collection of data. The questionnaire link was sent to respondents via emails and multiple social media platforms like Messenger®, and WhatsApp®. Participants were allowed 1 month (October 14, 2021, to November 14, 2021) to submit electronic responses, with reminders sent before the closing date. Medical students from multiple public and private sector medical institutes from all over Pakistan were approached. Electronically informed consent was received from each participant before initiating the survey and respondents could withdraw from the survey at any moment before submitting the response. The research was carried out in compliance with the Helsinki Declaration.

### Questionnaire development

After an extensive literature review, the questionnaire was drafted in English (2, 5, 8, 13). For professional insight on relevance, researchers and radiologists reviewed the draft. The final draft had 20 questions assessing the knowledge, interest, and exposure to IR. The demographic information included age, gender, and information about the medical college as a sector (public or private), location of the college, and year of study. Also, the questionnaire included information about future choice of medical career, either clinical or non-clinical medicine, self-reported knowledge of radiology in comparison to other medical specialties as excellent, good, fair, or poor, and choice of diagnostic radiology, IR, or any other medical specialty as a profession and experiencing an elective or mandatory IR rotation (2).

The following sections had questions about exposure to IR as the presence of the IR department in the institute, mandatory or elective IR rotation and duration of it, and factors to increase the exposure to IR and knowledge of participants about the procedures performed by interventional radiologists and the tools being employed (5, 8). Further, factors contributing to the choice of medical specialty and radiology as a career, the responsibilities of an interventional radiologist, and the prospects of IR were asked (8, 13).

### Data analysis

A descriptive analysis was conducted on collected data using Statistical Package for Social Sciences (SPSS) version 22.0 (IBM). Continuous variables were quantified as means and standard deviations, while categorical data were expressed using frequencies and percentages. The univariate analysis was used to analyze the association between dependent and independent variables. The potential factors influencing specialty selection were analyzed using the logistic regression model with a 95% confidence interval (95% CI), and a *p*-value < 0.05 was considered statistically significant.

## Results

### Demographics

A total of 570 responses were received with a response rate of 88% and a median age of 22 years, 60.7% were male, 58.1% were from a public institution, and 33.9% were from the 5^th^ year of medical school. 94.4% (538) opted for a future in the clinical specialty, of which 65.5% (373) chose radiology, with 28.8% (104) and 20.2% (115) preferred diagnostic radiology and IR, respectively. 59.6% (340) had poor to fair self-reported knowledge of radiology, and 83.5% (476) participants perceived IR with adequate to excellent prospects. 37.2% (212) of the respondent’s institutes did not have an IR department, and only 43.5% (248) of participants’ institutes had mandatory IR rotation, out of which 31% (177) had less than a 4-week rotation (Table 1).

**Table 1:** Baseline and demographic characteristics of the study participants.

Variables	Descriptive
Total	570 (100%)
Age	
Median (IQR)	22.0 (21.0–24.0)
Gender	
Male	346 (60.7%)
Female	224 (39.3%)
Institution sector	
Public	331 (58.1%)
Private	239 (41.9%)
The academic year of Medical School	
1^st^ year	89 (15.6%)
2^nd^ year	59 (10.4%)
3^rd^ year	80 (14.0%)
4^th^ year	149 (26.1%)
5^th^ year	193 (33.9%)
Career Path to be preferred	
Clinical (any specialty)	538 (94.4%)
Non-clinical (basic sciences)	32 (5.6%)
Self-reported knowledge of Radiology as compared to other fields	
Excellent	65 (11.4%)
Good	165 (28.9%)
Fair	223 (39.1%)
Poor	117 (20.5%)
If you have planned for Radiology as a career in the future, what would you prefer among these?	
Interventional Radiology	115 (20.2%)
Diagnostic Radiology	164 (28.8%)
Any one of the above two	94 (16.5%)
I will not choose radiology in any case	197 (34.6%)
Does your institute have an interventional radiology department?	
No	212 (37.2%)
Yes	337 (59.1%)
Do not know	21 (3.7%)
Self-reported knowledge of interventional radiology as a specialty	
Excellent	54 (9.5%)
Good	122 (21.4%)
Adequate	186 (32.6%)
Not knowledgeable	58 (10.2%)
Poor	150 (26.3%)
Duration of mandatory interventional radiology rotation in medical school	
1 week	73 (12.8%)
2 weeks	64 (11.2%)
3 weeks	40 (7.0%)
4 or more than 4 weeks	71 (12.5%)
None	322 (56.5%)
Duration of any elective radiology rotation if attended during medical school	
1 week	36 (6.3%)
2 weeks	51 (8.9%)
3 weeks	27 (4.7%)
4 or more than 4 weeks	52 (9.1%)
None	404 (70.9%)
If increased exposure to a clinical specialty increases the likelihood of making it a career choice, then how much exposure results in increased interest in that field?	
1 week	77 (13.5%)
2 weeks	132 (23.2%)
3 weeks	110 (19.3%)
4 or more than 4 weeks	251 (44.0%)
Willingness to attend interventional radiology rotation?	
No	133 (23.3%)
Yes	437 (76.7%)
Views about career prospects regarding Interventional Radiologists	
Excellent	148 (26.0%)
Good	214 (37.5%)
Adequate	114 (20.0%)
Poor	33 (5.8%)
Do not know	61 (10.7%)

### Relationship of baseline variables with those who categorically preferred careers in interventional radiology

Out of 115 participants, those above the age of 22 favored IR over participants under 22 (OR = 1.606, 95% CI = 1.057–2.439, *p*-value 0.026). Males preferred IR 1.5 times more than females (OR = 1.545, 95% CI = 0.999–2.390, *p*-value = 0.049), and 5^th^-year medical students preferred IR than other years of medical school (OR = 0.415, 95% CI = 0.242–0.711, *p*-value = 0.001). Participants willing to attend an IR rotation are more likely to prefer IR as a future medical specialty (OR = 3.464, 95% CI = 1.799–6.669, *p*-value = <0.001). Participants with an excellent view of IR’s future aspects showed 2.4 times more interest in IR as a career than those who did not know about IR (OR = 2.373, 95% CI = 1.109–5.080, *p*-value = 0.026; Table 2).

**Table 2:** Relationship of baseline variables with preferred careers in interventional radiology (*n* = 115).

Variables		Interventional radiology (*n* = 115)	May or may not I.R. (*n* = 455)	OR (CI = 95%)	*p*-value
Age	<22 years	46 (40.7%)	237 (52.4%)	-	0.026^*^
	>22 years	67 (59.3%)	215 (47.6%)	1.606 (1.057–2.439)	
Gender	Male	36 (31.3%)	188 (41.3%)	1.545 (0.999–2.390)	0.050^*^
	Female	79 (68.7%)	267 (58.7%)	-	
Institution sector	Public	49 (42.6%)	190 (41.8%)	-	0.869
	Private	66 (57.4%)	265 (58.2%)	1.035 (0.685–1.566)	
The academic year of Medical College	1^st^ year	13 (11.3%)	76 (16.7%)	0.388 (0.200–0.754)	0.005^*^
	2^nd^ year	6 (5.2%)	53 (11.6%)	0.257 (0.105–0.631)	0.003^*^
	3^rd^ year	14 (12.2%)	66 (14.5%)	0.482 (0.251–0.926)	0.028^*^
	4^th^ year	23 (20.0%)	126 (27.7%)	0.415 (0.242–0.711)	0.001^*^
	5^th^ year	59 (51.3%)	134 (29.5%)	-	-
Career Path to be preferred	Clinical	110 (95.7%)	428 (94.1%)	1.388 (0.522–3.687)	0.509
	Non-clinical	5 (4.3%)	27 (5.9%)	-	
Knowledge of Radiology	Excellent	15 (13.0%)	50 (11.0%)	1.650 (0.768–3.546)	0.199
	Good	46 (40.0%)	129 (38.9%)	1.535 (0.786–2.599)	0.178
	Fair	36 (31.3%)	177 (28.4%)	1.429 (0.823–2.863)	0.242
	Poor	18 (15.7%)	99 (21.8%)	-	-
Having an interventional radiology department in an affiliated institute	Yes	62 (55.4%)	275 (62.9%)	1.369 (0.899–2.084)	0.142
	No	50 (44.6%)	162 (37.1%)	-	
Knowledge of interventional radiology as a specialty	Excellent	11 (9.6%)	43 (9.5%)	1.343 (0.608–2.969)	0.466
	Good	30 (26.1%)	92 (20.2%)	1.712 (0.939–3.120)	0.079
	Adequate	44 (38.3%)	142 (31.2%)	1.627 (0.937–2.826)	0.084
	Not knowledgeable	6 (5.2%)	52 (11.4%)	0.606 (0.234–1.568)	0.302
	Poor	24 (20.9%)	126 (27.7%)	-	-
Mandatory interventional radiology rotation in medical school	Yes	38 (33.0%)	128 (28.1%)	1.261 (0.813–1.956)	0.300
	No	77 (67.0%)	327 (71.9%)	-	
How much exposure results in increased interest in a clinical specialty to make it a career choice?	1 week	11 (9.6%)	66 (14.5%)	0.542 (0.269–1.094)	0.088
	2 weeks	28 (24.3%)	104 (22.9%)	0.876 (0.527–1.458)	0.611
	3 weeks	17 (14.8%)	93 (20.4%)	0.595 (0.329–1.077)	0.086
	4 or more than 4 weeks	59 (51.3%)	192 (42.2%)	-	-
Willingness to attend interventional radiology rotation.	Yes	104 (90.4%)	333 (73.2%)	3.464 (1.799–6.669)	<0.001^*^
	No	11 (9.6%)	122 (26.8%)	-	
Views about career prospects regarding Interventional Radiologists	Excellent	47 (40.9%)	101 (22.2%)	2.373 (1.109–5.080)	0.026^*^
	Good	38 (33.0%)	176 (38.7%)	1.101 (0.513–2.362)	0.805
	Adequate	18 (15.7%)	96 (21.1%)	0.956 (0.411–2.225)	0.917
	Poor	2 (1.7%)	31 (6.8%)	0.329 (0.068–1.601)	0.169
	Do not know	10 (8.7%)	51 (11.2%)	-	-

* Statistically significant (*p*-value < 0.05).

### Participant’s responses regarding exposure and interest in the field

Regarding exposure to IR, 52.6 and 48.9% of participants considered ward rounds and attachment to the radiology department. Regarding the perspective toward the responsibilities of the interventional radiologist, participants responded that interventional radiologist treat major illnesses (43.3%) and do ward rounds (30.4%).Participants had good knowledge of tools used by interventional radiologists, as 45.8% correctly identified the tools as stents, micro-catheters, embolic materials, and needling technique tools (Table 3).

**Table 3:** Knowledge about interventional radiology and its tools.

Variables		Descriptive	Interventional radiology (*n* = 115)	May or may not I.R. (*n* = 455)	*p*-value
Which of the following will provide better student exposure to I.R.?	Ward rounds	300/570 (52.63%)	55/115 (47.8%)	245/455 (53.8%)	0.248
	Radiology department attachments	279/570 (48.94%)	71/115 (61.7%)	208/455 (45.7%)	0.002^*^
	Lecture from an interventional radiologist	127/570 (22.28%)	32/115 (27.8%)	95/455 (20.9%)	0.110
	Self-directed learning websites	60/570 (10.52%)	8/115 (7.0%)	50/455 (11.0%)	0.201
	Clinical research projects	111/570 (19.47%)	20/115 (17.4%)	91/455 (20.0%)	0.528
	Study modules	73/570 (12.80%)	15/115 (13.0%)	58/455 (12.7%)	0.932
	Multidisciplinary meetings	64/570 (11.22%)	18/115 (15.7%)	46/455 (10.1%)	0.093
If you are familiar with the word angioplasty, where did you gain exposure?	Cardiologist	244/570 (42.80%)	56/115 (48.7%)	188/455 (41.3%)	0.153
	Vascular Surgeon	70/570 (12.28%)	12/115 (10.4%)	58/455 (12.7%)	0.500
	General Surgeon	60/570 (10.52%)	10/115 (8.7%)	50/455 (11.0%)	0.474
	Interventional radiologist	55/570 (9.65%)	14/115 (12.2%)	41/455 (9.0%)	0.305
	Other	141/570 (24.73%)	23/115 (20.0%)	118/455 (25.9%)	0.188
Which of the following duties are provided by an Interventional Radiologist?	Ward rounds	173/570 (30.35%)	32/115 (27.8%)	141/455 (31.0%)	0.510
	Outpatient clinics	150/570 (26.31%)	28/115 (24.3%)	122/455 (26.8%)	0.592
	Admits patients	113/570 (19.82%)	21/115 (18.3%)	92/455 (20.2%)	0.638
	Treats patients with major illnesses	247/570 (43.33%)	32/115 (27.8%)	215/455 (47.3%)	<0.001^*^
	Treats patients with minor illnesses	123/570 (21.58%)	16/115 (13.9%)	107/455 (23.5%)	0.025^*^
	Does not treat patients	90/570 (15.79%)	21/115 (18.3%)	69/455 (15.2%)	0.416
Which of the following are the tools of interventional radiology?	Stents	50/570 (8.77%)	10/115 (8.7%)	40/455 (8.8%)	0.974
	Micro catheters	45/570 (7.89%)	7/115 (6.1%)	38/455 (8.4%)	0.421
	Embolic materials	55/570 (9.65%)	16/115 (13.9%)	39/455 (8.6%)	0.083
	Needling techniques like radiofrequency ablation, Micro-needling	117/570 (20.52%)	18/115 (15.7%)	99/455 (21.8%)	0.147
	All of above	261/570 (45.79%)	60/115 (52.2%)	201/455 (44.2%)	0.124
	None of above	42/570 (7.37%)	4/115 (3.5%)	38/455 (8.4%)	0.074

* Chi-square test.

### Participant’s awareness regarding procedures performed in IR

**Figure 1:**
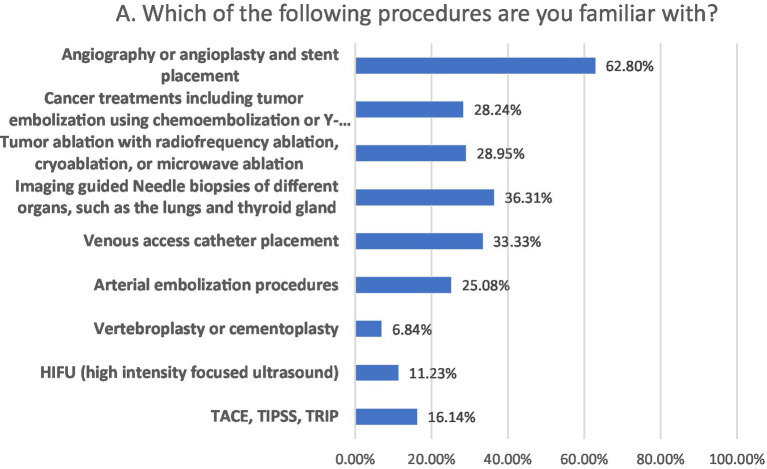
Participant’s awareness regarding procedures performed in IR.

Regarding procedures performed in IR, participants had familiarity with angioplasty/angiography (62.80), tumor embolization (28.24%), tumor ablation (28.95%), imaging-guided biopsies (36.31), venous catheter placement (33.33%), vertebroplasty (6.84%), high intensity focused ultrasound (HIFU; 11.23%) and trans-arterial chemo embolization (TACE), and trans-jugular intrahepatic portosystemic shunting (TIPSS; 16.14%; Figure 1).

### Factors influencing the choice of medical specialty

**Figure 2:**
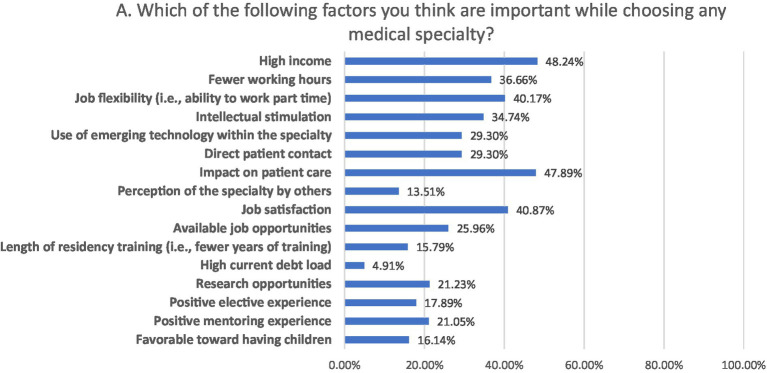
Participant’s awareness regarding procedures performed in IR.

The factors influencing participant’s choice of medical specialty included attractive salary (48.2%), impact on patient care (48.9%), job satisfaction (40.9%), job flexibility (40.2%), fewer working hours (36.7%), intellectual stimulation (34.7%), and direct patient care (29.3%; Figure 2).

### Factors influencing the choice of IR as a medical specialty

**Figure 3:**
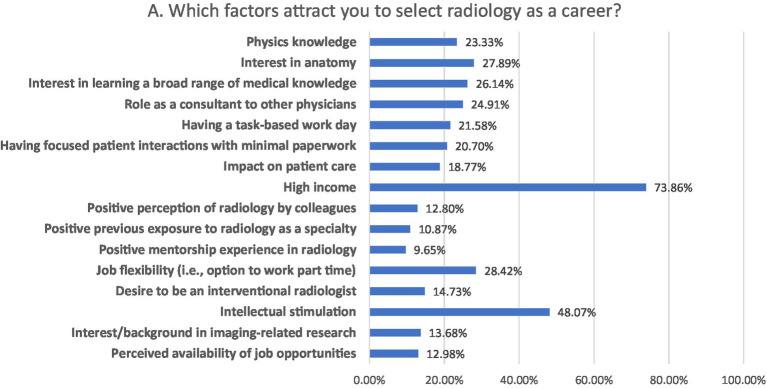
Factors influencing the choice of medical specialty.

The most significant factors influencing the choice of IR as a future medical specialty were high income (73.86%), intellectual stimulation (48.07%) and job flexibility (28.42%; Figure 3).

### Knowledge regarding procedures performed by interventional radiologists

**Figure 4:**
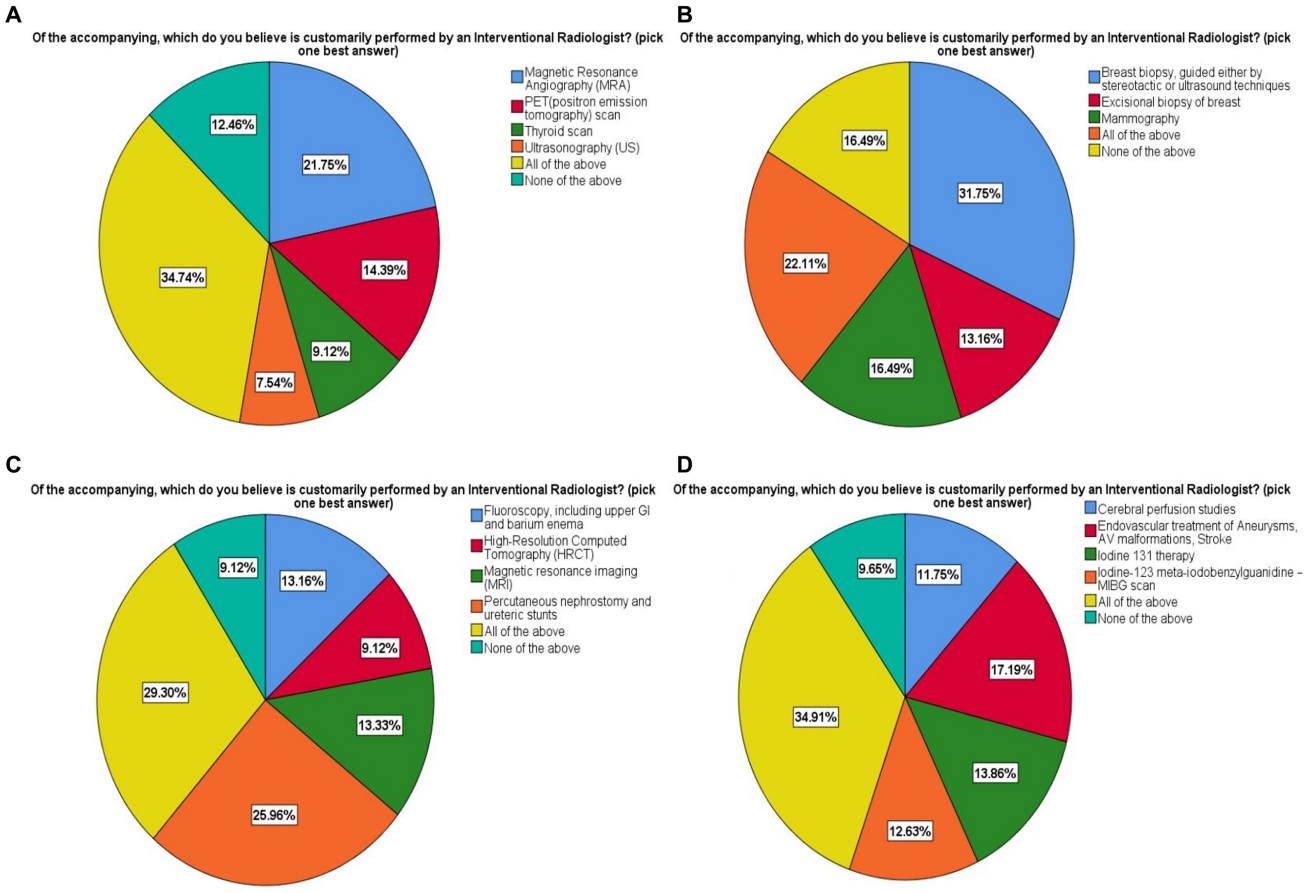
Knowledge regarding procedures performed by interventional radiologists. Shown are all the multiple choice questions with answers provided to choose from in all four components **(A–D)**.

The participants responded to the procedure performed by an interventional radiologist of the available options. In Figure 4A, magnetic resonance angiography (MRA; 21.75%) was the only procedure performed by IR of other available options including positron emission tomography (PET-CT; 14.39%), thyroid scan (9.12%), and ultrasound (7.45%). In Figure 4B, image-guided breast biopsy (31.75%) was the procedure performed by IR of the other provided interventions as excisional biopsy of the breast (13.16%) and mammography (16.49%). In Figure 4C, percutaneous nephrostomy and ureteric stents (25.96%) was the only intervention carried out by IR among the other interventions as fluoroscopy, upper GI and barium enema (13.16%), high resolution computed tomography (HRCT; 9.12%) and magnetic resonance imaging (MRI; 13.33%). In Figure 4D, endovascular treatment of aneurysmal malformations and stroke (29.30%) was the right answer of the procedure performed by IR among the others as cerebral perfusion studies, Iodine 131 therapy, and Iodine-132-metaiodobenzylguanidine scan (MIBG).

## Discussion

IR is facing unique challenges, such as low awareness levels and a need for qualified professionals, despite having immense potential and job security (2, 5, 8). This study assessed the level of knowledge, exposure, and interest in IR among medical students in Pakistan.

The majority, 94.4%, opted for a future in clinical specialty over basic sciences, which concord with a study conducted by Kim et al. that 87.7% of medical students intended a career in clinical medicine (4). 65.5% of the participants chose radiology, with 28.8 and 20.2% categorically interested in diagnostic radiology and IR, respectively. Sebastian et al. reported similar results with medical students in India interested in radiology (42%) and IR (36%) (5). In Europe, 41% considered IR, while in Saudi Arabia, 16.6 and 14.4% opted for diagnostic radiology and IR, respectively (2, 8). In the USA, 18.5% of 4^th^-year medical students chose radiology and did so on the advice of a mentor, faculty adviser, family member, or friend (13). Male gender has a significant association for preferring IR. Existing literature reports an association between male gender and a preference for procedural and technical-oriented medical specialties (10, 14).

63.5% had adequate to excellent self-reported knowledge of IR, which is in contrast with as 61.6% of respondents in India, 63% in Europe, and 52.4% in Oman reported a lack of awareness about IR (2, 5, 8). 37.2% of the participant’s institutes did not have an IR department with only 43.5% having mandatory IR rotation. Only 29% had attended elective IR rotations. Still, that is a small number compared to 76.7% being interested in attending IR rotations. Sebastian et al. reported that 58.7% of the students had experience with the IR department because IR services were available in 48% of the respondents’ institutions (5). Highlighting the fact that the presence of IR services in teaching hospitals would increase medical students’ exposure to and familiarity with IR. The knowledge and interest in IR as a career increased in medical students from 6 to 45% and 60 to 73%, respectively, after a 10-h educational session on IR (15).

Almost 62.8% of participants were familiar with angioplasty as an IR procedure, with cardiologists being the primary source (42.8%), which is consistent with earlier studies, as out of the 100% familiarity rate of angioplasty in India, 67.6% were made aware of angioplasty by cardiologist (5). In comparison, 83% were exposed to angioplasty by a European cardiologist (10). Recognition level of other procedures such as vertebroplasty (6.84%), HIFU (11.2%), and TACE, and TIPS (16.1%) was low, even though 45.8% of participants correctly identified tools used by an interventional radiologist. In contrast, Sebastian et al. reported only 9.2% familiarity with all of the IR procedures, with 74.2% being aware of neuro-interventional procedures as IR procedures (5). Participants responded to the interventional radiologist’s duties in the treatment of major illnesses (43.3%) and ward rounds (30.4%). Ward rounds are undertaken by IR specialists in 55 out of 97 departments (56.7%), according to a survey conducted by the European Society of Radiology (ESR) and the cardiovascular and interventional radiological society of Europe (CIRSE) (10). Regarding exposure to IR, participants considered ward rounds (56.2%) and attachment to the radiology department (48.9%). Leong et al. reported as 60% of European final-year medical students preferred interventional radiology rotation for exposure to the field. Direct exposure and hand on practice are considered the best learning mediums, making these the most suitable for educating medical students (8). 63.5% of respondents perceived IR. with good prospects accordant with that of Saudi Arabia (76%), and in Europe (80%) perceived IR. with good to excellent prospects (1, 2). Longer rotations (2 weeks to 4 or more weeks) in a clinical specialty raise the chance of choosing it as a career choice (2). The participants with a positive outlook on the prospects of IR were more interested in selecting IR as a career than those with poor knowledge and awareness of IR (2, 5, 8). Medical students with an excellent view of future aspects of IR had higher interest in IR as a career being consistent with Park et.al as the medical students being interested in IR were more certain about knowledge regarding IR (16).

Factors influencing the choice of IR as a future medical specialty were high income (73.86%), intellectual stimulation (48.07%) and job flexibility (28.42%). Park et al. reported that medical students interested in IR were more motivated by procedures (3.1/5), job market (2.8/5) and salary (2.6/5) and were less motivated by direct patient care (2.8/5) and longitudinal patient care (1.6/5) (16). The common factors influencing participants’ specialty selection were the attractive salaries, the effect on patient care, career prospects, personal interest, and job flexibility (5, 17). The major reasons for selecting radiology were high pay, intellectual stimulation, and an interest in anatomy. The most significant aspects alluded to were intellectual stimulation, work atmosphere, and influence on patient care (13, 18).

Our study emphasizes integrating the IR curriculum into undergraduate medical training. International organizations such as the CIRSE, the British Society of Interventional Radiology (BSIR), and the Indian Society for Vascular and Interventional Radiology (ISVIR) have devised various IR programs with yearly congresses like “Be Inspired” with hands-on workshops, interactive activities, and quiz programs customized specifically to introduce undergraduate medical students to IR (19, 20). Integrated regional support program (IRSP) is working in Pakistan to bring together specialists from various disciplines through a series of international conferences and local workshops with the goal of integration and knowledge management. The cardiology and endovascular surgery procedures overlap with interventional radiology procedures; it can be addressed if IR delivers higher qualitative expertise supported by research and technology than other specialties or by a multidisciplinary approach to treating patients (21, 22). Furthermore, a significant emphasis is to ensure that medical students have an understanding and convenient access to the training in the IR.

Radiology is a diverse and technical medical specialty incorporating all the latest innovations in modern sciences from virtual reality (VR) to artificial intelligence (AI). VR had been proven beneficial in IR training for learning and acquisition of procedural skills as angiography, angioplasty, stent placement and vascular catheterization (23). Furthermore, integration of AI techniques in IR also improves procedural planning and execution as well as facilitates follow up of treatment. AI would benefit individual patient management and also optimizes the radiology education globally (24). E-learning, VR and simulation platforms will raise the interest of medical students in IR and also improve their learning. Medical education should be updated with the latest innovations continuously to facilitate the training and education of medical students.

## Limitations

Our study has limitations. Firstly, the questionnaire was distributed electronically, which may have resulted in a selection bias. Secondly, because there were no responses from some medical colleges, it may not reflect all the medical students in the country. Thirdly, the study used a survey-based approach, which may have introduced a certain degree of response bias because respondents interested in the subject were more likely to fill out the questionnaire correctly. The response to self-reported knowledge about IR was subjective by the individual participants resulting in the biased and subjectivity of responses. In fact, in this study medical students’ self-assessment of their knowledge about IR is only their perception of their knowledge and not their actual knowledge about IR as examined in detail by Park et al. (16).

## Conclusion

IR is a nascent field in Pakistan hampered by a personnel shortage and the need for more awareness among medical students. This study evaluated exposure, understanding, and interest in IR among Pakistani medical students. The majority were inclined towards the clinical side of medicine, with many interested in radiology and IR. Participants reported gaps in knowledge, understanding of IR, and limited exposure to during medical school. A structured, integrated undergraduate IR medical curriculum with exposure to techniques, workflow and procedures may improve the prospects of IR in medical students. Teaching institutes with IR departments should offer elective programs even to the medical students of other institutes. Regardless of their potential specialty, all undergraduates should have basic knowledge regarding IR. More IR departments should be established in teaching institutes, and training slots should be available to increase the number of IR professionals to overcome deficiencies in healthcare.

## Data availability statement

The raw data supporting the conclusions of this article will be made available by the authors, without undue reservation.

## Ethics statement

The studies involving humans were approved by Informed consent was obtained from all the participants and also from parents/legal guardians of minors and illiterates. The Foundation University, Rawalpindi, Pakistan approved the study protocol (FF/FUMC/215-192 PHY/22). We confirm that all methods were carried out in accordance with relevant guidelines and regulations. Participants were assured about the confidentiality of any obtained information. The responses were kept confidentially and data from this research was managed only researchers in this study, Results will be used only for research and data cannot be traced back to their original sources. The studies were conducted in accordance with the local legislation and institutional requirements. Written informed consent for participation in this study was provided by the participants’ legal guardians/next of kin.

## Author contributions

MT, MA, and MuC: conceived the idea. MuC, MaC, SS, and MT collected the data. ME and MA analyzed and interpreted the data. MuC, WT, MaC, SS, MM, ZY, and MT did write up of the manuscript. ZY, AZ, ME, and MA reviewed the manuscript for intellectual content critically. All authors contributed to the article and approved the submitted version.

## Abbreviations

IR: Interventional radiology
TIPSS: Trans jugular intra-hepatic Porto-systemic shunt
TACE: trans-arterial chemo-embolization
CIRSE: Cardiovascular and Interventional Radiological Society of Europe
ISVIR: Indian society of vascular and interventional radiology
IRSP: Interventional radiology society of Pakistan
BSIR: British Society of Interventional Radiology
ESR: European Society of Radiology
